# Prediction Model for the Clearance of Hepatitis B Surface Antigen in Patients with Chronic Hepatitis B before Interferon Therapy: A Prospective Case–Control Study

**DOI:** 10.3390/diagnostics14010118

**Published:** 2024-01-04

**Authors:** Nan Geng, Lina Ma, Yi Jin, Junfeng Lu, Yanhong Zheng, Junli Wang, Xiaoxiao Wang, Xinyue Chen

**Affiliations:** Beijing Youan Hospital, Capital Medical University, Beijing 100069, China; 0960106@163.com (N.G.); malina_bj@126.com (L.M.); 13811056403@126.com (Y.J.); junfeng_1980@aliyun.com (J.L.); gjyl115@126.com (Y.Z.); yinzhixiangmu@126.com (J.W.); wxxdoc@163.com (X.W.)

**Keywords:** chronic hepatitis B, interferon-α, HbsAg clearance, prediction model, prospective case–control study

## Abstract

To evaluate the prediction model comprised of patients’ laboratory results and single-nucleotide polymorphism (SNP) markers of host gene for the clearance of hepatitis B surface antigen (HBsAg) in patients with chronic hepatitis B (CHB) who underwent interferon (IFN)-α therapy, this prospective case–control study enrolled 131 patients with CHB who underwent IFN-α-based regimens in our hospital between January 2015 and September 2019. Among them, 56 cases were without HBsAg clearance, while the other 75 cases had HBsAg clearance. Multivariable logistic regression analysis showed that CYP27B1 rs4646536 (odd ratio [OR] = 0.155, 95% CI: 0.030–0.807, *p* = 0.027), PAK4 rs9676717 (OR = 11.237, 95% CI: 1.768–71.409, *p* = 0.010), IL28B rs12979860 (OR = 0.059, 95% CI: 0.006–0.604, *p* = 0.017), baseline HBsAg (OR = 0.170, 95% CI: 0.040–0.716, *p* = 0.016), and HBeAg status (OR = 3.971, 95% CI: 1.138–13.859, *p* = 0.031) were independently associated with HBsAg clearance. The model that included rs3077, rs4646536, rs9676717, rs2850015, rs12979860, baseline HBsAg, HBeAg status, and HBV DNA had the best prediction performance for HBsAg clearance prediction, with AUC = 0.877, 80% sensitivity, and 81% specificity. In conclusion, laboratory results and gene polymorphisms before treatment might have a good predictive value for HbsAg clearance after IFN-α treatment in CHB.

## 1. Introduction

Approximately 296 million patients are living with chronic hepatitis B virus (HBV) in the world, with prevalence ranging from <2% in the United States and other western countries to over 5% in some parts of East Asia, Southeast Asia, and sub-Saharan Africa [1,2,3]. Chronic HBV infection can be classified into five phases: (1) HbeAg-positive chronic infection, (2) HbeAg-positive chronic hepatitis, (3) HbeAg-negative chronic infection, (4) HbeAg-negative chronic hepatitis, and (5) HBsAg-negative phase [4]. The goal for the treatment of CHB is achieving a clinical or functional cure (i.e., continuous virological response and hepatitis B surface antigen (HBsAg) negative conversion or with anti-HBs positive conversion, normal ALT, and slight or no liver tissue lesions), which can control the progression of CHB and reduce the related complications and death [4,5,6].

Currently, interferon-α (IFN-α) and nucleoside (acid) analogs (NUCs) are the primary antiviral drugs that can significantly improve the prognosis of CHB [3,4]. IFN treatment is an important antiviral method. Unfortunately, few patients achieve functional cures through antiviral treatment. The HBsAg clearance rate of IFN-α is relatively high compared with NUCs [5,6]. Therefore, IFN-α treatment is the most promising method for HBsAg clearance and CHB clinical treatment but some patients are not responding [5,6]. Therefore, the cure rate could be improved by using an adequate selection of the most likely patients to respond. Several studies showed that host genes, alanine aminotransferase (ALT) levels, HBV genotypes, HBV-DNA quantification, anti-HBC, and HBsAg levels are important predictors of antiviral efficacy [7,8,9]. These predictors can help improve the clinical cure rate but the results are inconsistent [10,11,12].

Since implementing the Human Genome Project (HGP), host factors have become the focus of research on the occurrence of various diseases and drug efficacy. There are many reports on the progression, prognosis, and host factors related to the treatment of CHB [13,14,15]. The vital role of host gene polymorphism in the efficacy response of CHB patients to IFN-α is also recognized [16,17,18]. Nevertheless, although there are numerous studies on the subject, most of them do not examine a functional cure but focus on HBV DNA negative conversion and HBeAg seroconversion. Moreover, the conclusions on the association between gene polymorphisms and treatment efficacy are also inconsistent [19,20,21].

Disease outcomes are related to viruses, environment, and host factors [22,23]. In addition, age, sex, alcohol, obesity, diabetes, renal failure, and host gene variation can also affect the clinical course of HBV infection [24,25,26]. Therefore, this study aimed to evaluate the prediction models comprised of patients’ laboratory results and the single-nucleotide polymorphism (SNP) markers of the host gene for the clearance of hepatitis B surface antigen (HBsAg) in patients with chronic hepatitis B (CHB) undergoing interferon (IFN)-α therapy.

## 2. Materials and Methods

### 2.1. Study Design and Participants

This prospective case–control study enrolled Chinese Han patients with CHB treated at the Beijing Youan Hospital and collected their blood samples for laboratory and gene detection between January 2015 and September 2019. The study protocol was approved by the ethics committee of the Beijing Youan Hospital. Written informed consent was obtained from all participants.

The Inclusion criteria were: (1) 18–70 years of age, (2) HBV DNA ≥ 2000 IU/mL, (3) ALT ≥ 1 × the upper limit of normal value (ULN), and (4) diagnosis of CHB according to the Chinese Guideline for the Prevention and Treatment of Chronic Hepatitis B [6]. The exclusion criteria were: (1) known history of allergy or contraindications to IFN-α products, (2) complicated with serious diseases or malignant tumor, (3) epilepsy or other central nervous system dysfunction, (4) decompensated cirrhosis, liver cancer, history of mental illness, autoimmune diseases, accompanied by severe infection, retinal diseases, chronic obstructive pulmonary disease, thyroid diseases, etc., (5) complicated with other infectious diseases, (6) preparing for pregnancy, pregnancy, or breast-feeding, (7) neutrophil count before IFN-α treatment <1.5 × 10^9^/L and/or platelet count <90 × 10^9^/L, (8) during IFN-α treatment, use of chemotherapy, traditional Chinese medicine, or immune preparations (such as glucocorticoid, thymic peptide, thymus pentapeptide, thymus method, etc.), (9) incomplete course of treatment, or (10) lost to follow-up.

In order to achieve no statistical difference in gender and age between the two groups, we matched the two groups of patients. After matching by sex and age, 131 participants were enrolled: 56 without HBsAg clearance (non-clearance group) and 75 with HBsAg clearance (HbsAg clearance group). 

### 2.2. Procedures

Patients with a treatment course of 48–96 weeks based on IFN-α with or without oral NUCs (including entecavir (ETV) 0.5 mg/day or adefovir dipivoxil 10 mg/day) were screened. IFN-α includes long-acting PEG-IFN-α 135 μg/week (PEG-IFN-α2a or PEG-IFN-α2b) or IFN-α 50 μg qd (IFN-α1b, Beijing Sanyuan gene Pharmaceutical Co., Ltd. Beijing, China). The participants’ medical records were extracted and entered into the electronic data acquisition system of this study (Empower EDC, X&Y Solutions Inc., Beijing, China). This study recorded: (1) the basic information of the participants, such as name, sex, age, and location, (2) drug information, including classification, dose, usage, and prescription times of IFN-α and NUCs, and (3) laboratory results before and after participants were treated with IFN-α class drugs. HBsAg (IU/mL), HBeAg (IU/mL), HBV DNA (IU/mL), and ALT (U/L) were measured every 3 months.

The participants were divided into the HBsAg clearance and non-clearance groups according to the occurrence of HBsAg clearance due to IFN-α treatment or not within 96 weeks. HBsAg clearance was defined as antiviral therapy achieved HBsAg clearance or conversion (accompanied by anti-HBs production), hepatitis B e-antigen (HBeAg) serum conversion or staying negative, HBV DNA lower than the lower limit of detection value, and ALT < 1 × ULN before 96 weeks of treatment and maintained for 24 weeks.

### 2.3. Host Genotyping

Clinical blood samples were processed using the gDNA Extraction Kit (Beijing Tiangen Biotechnology Co., Ltd., Beijing, China) according to the kit’s instructions to extract genomic DNA from 400 μL of blood/case. Proteinase K solution (20 μL) was added, fully mixed, and incubated at 56 °C for 10 min. The final product was dissolved in 120 μL of eluent. The DNA concentration and purity were detected using agarose gel electrophoresis. The OD260/OD280 value was detected using a micro-volume ultraviolet spectrophotometer (FC-1100, Hangzhou Life Biotech Co., Ltd. Hangzhou, China). The DNA concentration was adjusted to 30 ng/μL as a template for detecting SNPs using mass spectrometry. The detected polymorphisms were IL28B rs12980275, MxA-123 rs17000900, IL10-592 rs1800872, IPS1 rs2464, HLA-DPA1 rs3077, CYP27B1 rs4646536, MxA rs469083, STA T4 rs7574865, IL28B rs8099917, HLA-DPB1 rs9277535, PAK4 rs9676717, IFNAR1 rs2850015, and IL28B rs12979860. The genotypes of these 13 genes were performed using the Sequenom Mass Array platform according to the manufacturer’s instructions.

### 2.4. Statistical Analysis

SPSS 22.0 (IBM Corp., Armonk, NY, USA) and Empowerstats (X&Y Solutions, Inc.) were used for statistical analysis. Continuous data were expressed as mean ± standard deviation (SD). Categorical data were expressed as n (%). The frequencies of the 13 SNPs in the HBsAg clearance and non-clearance groups were compared using the Chi-square test. The differences in baseline results between the two groups were determined using Student’s t-test or the Mann–Whitney U-test. More than two groups were compared by one-way ANOVA. The deviation between each SNP and the Hardy–Weinberg equilibrium was evaluated using the Fisher exact test (https://ihg.gsf.de/cgi-bin/hw/hwa1.pl accessed on 5 January 2022). Multivariable logistic regression analysis and receiver operating characteristic curve (ROC) were used to establish a prediction model. *p*-values, odds ratios (ORs), and 95% confidence intervals (CIs) were calculated. The area under the ROC curve (AUC) was used to determine the most accurate predictor of HBsAg clearance. Two-sided *p*-values < 0.05 were considered statistically significant.

## 3. Results

Finally, 131 participants were enrolled: 56 without HBsAg clearance (non-clearance group) and 75 with HBsAg clearance (HbsAg clearance group). The HBV DNA log (IU/mL) (*p* = 0.006), baseline HbsAg (IU/mL) (*p* = 0.003), and HbeAg status (*p* < 0.001) showed significant differences between the two groups (Table 1).

The deviation between each SNP and Hardy–Weinberg equilibrium was evaluated. The results showed that IL28B rs12980275, MxA-123 rs17000900, IL10-592 rs1800872, IPS1 rs2464, HLA-DPA1 rs3077, CYP27B1 rs4646536, MxA rs469083, STAT4 rs7574865, IL28B rs8099917, HLA-DPB1 rs9277535, PAK4 rs9676717, IFNAR1 rs2850015, and rs12979860 had no deviation from equilibrium.

**Figure 1:**
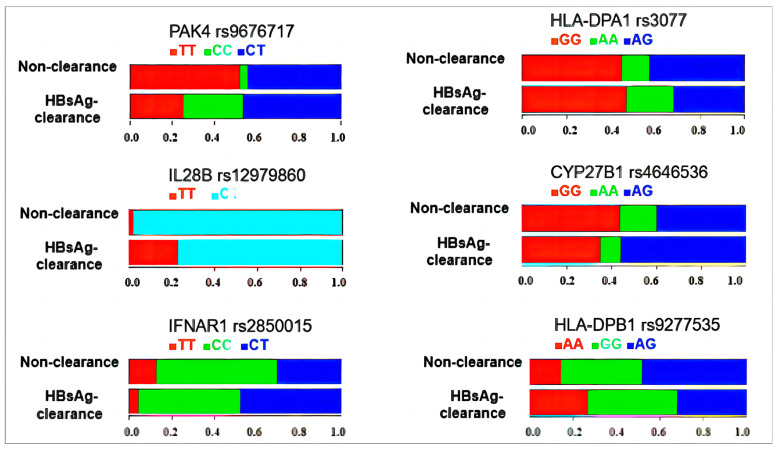
Polymorphism distributions in the HBsAg clearance and non-clearance groups. For most polymorphisms, there were frequencies of three genotypes in each group, with a sum of 1.0 (100%). For IL28B 12979860, there were two genotypes in each group, also with a sum of 1.0 (100%). Different colors indicate the frequencies of the genotypes for a given polymorphism.

The polymorphism frequency analysis showed that the frequencies of the PAK4 rs9676717 TT, CC, and CT genotypes in the HBsAg clearance group were 25.3%, 28.0%, and 46.7%, respectively, and those in the non-clearance group were 51.8%, 3.6%, and 44.6%, respectively. The frequency of PAK4-CT in the two groups was similar. The frequency of CC in the HBsAg clearance group was significantly higher (HBsAg responders preferred) (*p* < 0.05), and the frequency of TT was significantly lower (HBsAg responders preferred) (*p* < 0.05). PAK4 rs9676717 CC vs. TT had a *p* < 0.001. In addition, the IL28B rs12979860 TT, IFNAR1 rs2850015 CT, and HLA-DPB1 rs9277535 AA genotypes were the most preferred genotypes for a response, and the HBsAg clearance rate was higher than in the control group (IL28B rs12979860 TT vs. CT, *p* < 0.001; IFNAR1 rs2850015 CT vs. TT, *p* = 0.024; HLADPB1 rs9277535 AA vs. AG, *p* = 0.037). There were no correlations between the SNPs in the other nine genes and the responses to IFN-α (Table 2 and Figure 1).

The multivariable logistic regression analysis showed that CYP27B1 rs4646536 (odd ratio [OR] = 0.155, 95% CI: 0.030–0.807, *p* = 0.027), PAK4 rs9676717 (OR = 11.237, 95% CI: 1.768–71.409, *p* = 0.010), IL28B rs12979860 (OR = 0.059, 95% CI: 0.006–0.604, *p* = 0.017), baseline HBsAg (OR = 0.170, 95% CI: 0.040–0.716, *p* = 0.016), and HBeAg status (OR = 3.971, 95% CI: 1.138–13.859, *p* = 0.031) were independently associated with the IFN-α, which induced the HBsAg clearance rate in 48–96 weeks (Table 3).

**Figure 2:**
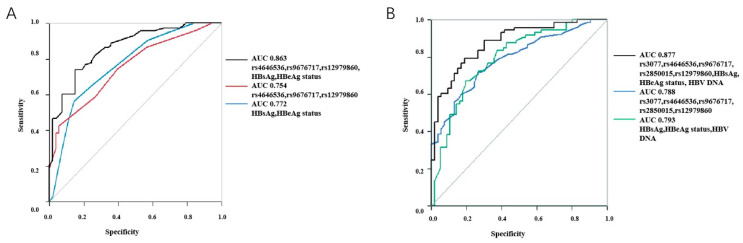
The prediction model was analyzed by logistic regression and shown by the receiver operating characteristics (ROC) curve in predicting responses of chronic hepatitis B (CHB) patients treated with interferon (IFN)-α. (**A**) The combination of potential factors from the multivariable analysis was analyzed by logistic regression, and the accuracy was shown using a ROC curve. (**B**) More factors displaying the differences in clinical characteristics and polymorphism analysis were incorporated into the logistic regression, and the prediction effect is shown using a ROC curve.

The combination of these independent risk factors from the multivariable analysis was analyzed using the ROC analysis. The results showed that the AUC was 0.863, sensitivity was 0.740, and specificity was 0.849, which had a better prediction performance than SNPs only (AUC = 0.788, sensitivity = 0.720, and specificity = 0.736) or laboratory results only (AUC = 0.793, sensitivity = 0.671, and specificity = 0.804) (Table 4 and Figure 2A).

Different models of laboratory results and gene polymorphism were analyzed using the ROC analysis. The model including rs3077, rs4646536, rs9676717, rs2850015, rs12979860, baseline HBsAg, HBeAg status, and HBV DNA displayed the best prediction performance (AUC = 0.877, sensitivity = 0.795, and specificity = 0.811), which showed a similar prediction performance (AUC = 0.877, sensitivity = 0.753, and specificity = 0.849) as rs9277535 was added to the model. When rs3077 was excluded from the model, the prediction performance slightly decreased (AUC = 0.876, sensitivity = 0.781, and specificity = 0.830) (Table 4 and Figure 2B).

## 4. Discussion

This study showed that the laboratory results and gene polymorphisms before treatment might have a good predictive value for HBsAg clearance after IFN-α treatment in patients with CHB. [23,24] Many host gene mutations, such as human leukocyte antigen (HLA), cytokine and chemokine, toll-like receptor (TLR), microRNA, and vitamin D-related genes, were found to affect the outcome of HBV infection [15,16,17]. Theoretically, polymorphisms in any gene encoding proteins (receptors, enzymes, cytokines, etc.) involved closely or not with the mechanisms of HBV infection of healthy cells, replication, host immunity, etc., are likely to affect the prognosis of CHB.

IFN-α is part of the backbone for the management of CHB [3,4]. Although the HBsAg clearance rate is high with IFN-α treatment, treatment failures can be observed [5,6]. Polymorphisms associated with the IFN signaling pathway can potentially affect patients’ response to IFN-α [16,17,18]. Qi et al. [16] showed that STAT4 genetic polymorphism significantly affected HBeAg seroconversion in HBeAg-positive chronic hepatitis B patients receiving PEG-IFN-α therapy. Wu et al. [17] showed that CYP27B1 polymorphisms are associated with IFN-α efficacy in HBeAg-positive patients. A pilot study by King et al. [18] identified two SNPs in the IFN pathway that were associated with the response to IFN therapy in patients with CHB. Many studies evaluated the relationship between IFN-α treatment results and host genetics and revealed a potential correlation between IFN-α treatment and HLA, STAT4, vitamin D-related genes, and some ISGS, as reviewed by Zhang et al. [27], but the exact loci remain unknown. In the present study, 13 gene loci were analyzed at the same time to establish an accurate prediction model to identify patients who could achieve a clinical cure. In the present study, the baseline HBV DNA levels, baseline HBsAg levels, and baseline HBeAg status were associated with HBsAg loss, as supported by the guidelines [4,5,6].

Interestingly, it was found that PAK4 rs9676717 CC, IL28B rs12979860 TT, IFNAR1 rs2850015 CT, and HLA-DPB1 rs9277535 AA were the genotypes associated with the HBsAg response in the patient population. PAK4 is a serine/threonine p21-activated kinase gene located about 64 kb upstream of the IL-28B gene [28]. PAK4 regulates cytoskeleton remodeling and affects directional movement, invasion, metastasis, and growth [28]. The rs9676717 SNP in PAK4 was independently associated with the response to IFN in Chinese patients with CHB [29]. The IL-28B gene encodes IFN-λ3 and plays an antiviral role by activating the JAK-STAT signal pathway [30]. IL28B rs12979860, rs8099917, and rs12980275 yielded conflicting results in predicting HBeAg seroconversion in patients treated with PEG-IFN-α-2a or PEG-IFN-α-2b [31,32]. IFNAR1 encodes the type I IFN receptor (IFN-αR1) that binds IFN-α and induces antiviral protein synthesis [33]. A polymorphism in the promoter of IFNAR1 and another polymorphism in the coding region and linkage disequilibrium with the first are involved in the genetic susceptibility to chronic infection by HBV [34]. HLA plays an important role in viral protein recognition and adaptive immune regulation [35]. IFN-α can upregulate HLA and regulate the adaptive immunity against HBV [36]. CYP27B1 is a vitamin D-related gene and can enhance IFN-α-mediated activation of the JAK-STAT pathway and increase the expression of antiviral proteins [37]. Therefore, these genes are involved in the host and treatment responses to CHB, and any polymorphisms that influence their expression or activity can probably influence the host and treatment responses to IFN-α in patients with CHB.

The multivariable logistic regression analysis identified that the potential factors, including CYP27B1 rs4646536, PAK4 rs9676717, IL28B rs12979860, baseline HBsAg, and HBeAg status, were independently associated with the IFN-α-induced HBsAg clearance. The AUC of the ROC curve was 0.863, indicating a high predictive value. In order to improve the accuracy of regression, laboratory and genetic factors were combined, and the combination of rs3077, rs4646536, rs9676717, rs2850015, rs12979860, HBsAg baseline level, HBeAg status, and HBV DNA baseline level were included; the highest AUC was 0.877. rs3077 contributed to the prediction (the AUC increased from 0.876 to 0.877), while rs9277535 did not change the AUC. Future and more refined models should also include clinical and miRNA parameters in addition to the genetic ones. Indeed, Zhang et al. [38] showed that a model based on 11 response-related miRNAs could predict the response to IFN-α in patients with CHB. Another model was built by Tan et al. [39] using 11 other miRNAs. Age, sex, ALT levels, bile acids, obesity, insulin resistance, alcohol consumption, and anti-IFN antibodies are also involved in the response to IFN-α in patients with CHB [40]. Future models should try integrating all these parameters.

This study has limitations. The results were obtained from a limited sample of participants since the study involved only one hospital. Only 13 SNPs were tested after selection based on genes susceptible to being involved in IFN signaling, but several additional SNPs can probably be associated with the response to IFN-α in patients with CHB. 

In our study, in order to obtain the matching of the HBsAg clearance group and the non-clearance group in order to obtain interferon-sensitive genes with the gene polymorphism related to the interferon pathway, matching screening of the two groups of patients increased the rate of HBsAg clearance to some extent.

In future studies, we will expand the research sample, increase the proportion of the control group after obtaining more HBsAg clearance patients, and further obtain the research results on interferon-sensitive genes. Such future studies would then succeed in improving the predictive model.

## 5. Conclusions

In conclusion, this study suggests that it is possible to make a preliminary prediction of the response to IFN-α in patients with CHB based on host gene variants (rs3077, rs4646536, rs9676717, rs2850015, and rs12979860) and laboratory results (HBsAg, HBeAg, and HBV DNA). These findings might provide a basis for a personalized and more precise treatment of patients with CHB.

## Figures and Tables

**Table 1:** Characteristics of the participants.

Characteristics		Non-Clearance, n = 56	HBsAg Clearance, n = 75	*p*
Age, mean ± SD		41.5 ± 10.8	40.5 ± 11.1	0.590
Sex, n (%)	Male	28 (50)	45 (60)	0.250
	Female	28 (50)	30 (40)	
ALT (U/L), mean ± SD		77.5 (105)	64.8 (71.7)	0.410
ALT elevation, n (%)	Yes	37 (67.3)	56 (74.7)	0.36
	No	18 (32.7)	19 (25.3)	
HBV DNA Log (IU/mL), n (%)	n ˃ median	29 (51.8)	21 (28.0)	0.006
	n ≤ median	27 (48.2)	54 (72.0)	
Baseline HBsAg (IU/mL), n (%)	≤99	4 (7.1)	21 (28.0)	0.003
	100–1000	20 (35.7)	29 (38.7)	
	1001–2000	9 (16.1)	12 (16.0)	
	˃2000	23 (41.1)	13 (17.3)	
HBeAg status, n (%)	Baseline negative	36 (64.3)	45 (60.0)	<0.001
	Seroconversion	11 (19.6)	30 (40.0)	
	No seroconversion	9 (16.1)	0 (0.0)	

ALT: alanine aminotransferase; HBeAg: hepatitis B e-antigen; HBsAg: hepatitis B surface antigen; SD: standard deviation.

**Table 2:** Polymorphism frequencies in HBsAg clearance and non-clearance group.

Polymorphism		Polymorphism Distribution			Odd Ratio, 95% CI	*p*
		Non-Clearance, n = 56	HBsAg Clearance, n = 75			
PAK4, rs9676717, n (%)	TT	29 (51.8)	19 (25.3)	CC vs. TT	16.026, 3.36–76.38	<0.001
	CC	2 (3.6)	21 (28.0)			
	CT	25 (44.6)	35 (46.7)			
IL28B, rs12979860, n (%)	TT	1 (1.9)	17 (22.7)	TT vs. CT	15.534, 1.998–120.777	<0.001
	CT	53 (98.1)	58 (77.3)			
IFNAR1, rs2850015, n (%)	TT	7 (12.5)	3 (4.0)	CT vs. TT	4.940, 1.136–21.5	0.024
	CC	32 (57.1)	36 (48.0)			
	CT	17 (30.4)	36 (48.0)			
HLA-DPB1, rs9277535, n (%)	AA	8 (14.3)	20 (26.7)	AA vs. AG	2.812, 1.048–7.548	0.037
	GG	21 (37.5)	31 (41.3)			
	AG	27 (48.2)	24 (32.0)			
CYP27B1, rs4646536, n (%)	GG	24 (43.6)	26 (34.7)	AG vs. AA	2.455, 0.805–7.480	0.108
	AA	9 (16.4)	7 (9.3)			
	AG	22 (40.0)	42 (56.0)			
HLA-DPA1, rs3077, n (%)	GG	25 (44.6)	35 (46.7)	AA vs. AG	2.286, 0.797–6.552	0.120
	AA	7 (12.5)	16 (21.3)			
	AG	24 (42.9)	24 (32.0)			
IL28B, rs12980275, n (%)	AA	50 (89.3)	59 (78.7)	AG vs. AA	2.119, 0.765–5.869	0.143
	GG	0	1 (1.3)			
	AG	6 (10.7)	15 (20.0)			
IL28B, rs8099917, n (%)	GG	0	1 (1.3)	GT vs. TT	2.119, 0.765–5.869	0.143
	TT	50 (89.3)	59 (78.7)			
	GT	6 (10.7)	15 (20.0)			
IL10-592, rs1800872, n (%)	TT	18 (32.7)	31 (41.3)	TT vs. GT	1.670, 0.783–3.562	0.183
	GG	5 (9.1)	11 (14.7)			
	GT	32 (58.2)	33 (44.0)			
MxA, rs469083, n (%)	TT	6 (10.7)	5 (6.7)	CT vs. TT	1.548, 0.745–3.219	0.241
	CC	29 (51.8)	33 (44.0)			
	CT	21 (37.5)	37 (49.3)			
STAT4, rs7574865, n (%)	GG	31 (55.4)	36 (48.0)	GT vs. GG	1.312, 0.632–2.724	0.466
	TT	4 (7.1)	7 (9.3)			
	GT	21 (37.5)	32 (42.7)			
MxA-123, rs17000900, n (%)	AA	1 (1.8)	3 (4.1)	AA vs. CC	2.423, 0.243–24.154	0.437
	CC	42 (75.0)	52 (70.3)			
	AC	13 (23.2)	19 (25.7)			
IPS1, rs2464, n (%)	CC	31 (56.4)	42 (56.0)	TT vs. CC	1.107, 0.288–4.260	0.882
	TT	4 (7.3)	6 (8.0)			
	CT	20 (36.4)	27 (36.0)			

**Table 3:** Multivariable analysis of factors associated with responses in CHB patients treated with IFN-α.

Characteristics	OR (95% CI)	*p*
HLA-DPA1, rs3077	1.308 (0.264–6.492)	0.743
CYP27B1, rs4646536	0.155 (0.030–0.807)	0.027
PAK4, rs9676717	11.237 (1.768–71.409)	0.010
IFNAR1, rs2850015	2.353 (0.297–18.641)	0.418
IL28B, rs12979860	0.059 (0.006–0.604)	0.017
HLA-DPB1, rs9277535	0.696 (0.140–3.469)	0.659
HBsAg, (IU/mL),	0.170 (0.040–0.716)	0.016
HBeAg status	3.971 (1.138–13.859)	0.031
HBV DNA, Log (IU/mL)	0.843 (0.648–1.096)	0.202

OR: odds ratio; CI: confidence interval; ALT: alanine aminotransferase; HBeAg: hepatitis B e-antigen; HBsAg: hepatitis B surface antigen; HBV: hepatitis B virus.

**Table 4:** Comparison of prediction models analyzed by logistic regression and shown by AUC, sensitivity, specificity, TP, FP, FN, and TN in predicting responses of CHB patients treated with IFN-α.

Factors	AUC	Sensitivity	Specificity	TP (a)	FP (b)	FN (c)	TN (d)
rs3077, rs4646536, rs9676717, rs2850015, rs12979860, baseline HBsAg, HBeAg status, baseline HBV DNA	0.877	0.795	0.811	58	10	15	43
rs9277535, rs3077, rs4646536, rs9676717, rs2850015, rs12979860,	0.877	0.753	0.849	55	8	18	45
baseline HBsAg, HBeAg status, baseline HBV DNA							
rs4646536, rs9676717, rs2850015, rs12979860, baseline HBsAg, HBeAg status, baseline HBV DNA	0.876	0.781	0.830	57	9	16	44
rs4646536, rs9676717, rs12979860, baseline HBsAg, HBeAg status	0.863	0.740	0.849	54	8	19	45
Baseline HBsAg, HBeAg status, baseline HBV DNA	0.793	0.671	0.804	49	11	24	45
rs3077, rs4646536, rs9676717, rs2850015, rs12979860	0.788	0.720	0.736	54	14	21	39
Baseline HBsAg, HBeAg status	0.772	0.562	0.857	41	8	32	48
rs4646536, rs9676717, rs12979860	0.754	0.427	0.943	32	3	43	50

AUC: area under the curve; TP: true positive; TN: true negative; FP: false positive; FN: false negative.

## Data Availability

Data available on request due to restrictions eg privacy or ethical.
